# Air quality in post-mining towns: tracking potentially toxic elements using tree leaves

**DOI:** 10.1007/s10653-022-01252-6

**Published:** 2022-03-25

**Authors:** Fabrizio Monaci, Stefania Ancora, Luca Paoli, Stefano Loppi, Jürgen Franzaring

**Affiliations:** 1 Department of Life Sciences, University of Siena, Via Mattioli 4, Siena, Italy; 2 Department of Physical Sciences, Earth and Environment, University of Siena, Via Mattioli 4, Siena, Italy; 3 Department of Biology, University of Pisa, Via Luca Ghini, 13, 56126 Pisa, Italy; 4 Institute of Landscape and Plant Ecology, University of Hohenheim, Ottilie-Zeller-Weg 2, 70599 Stuttgart, Germany

**Keywords:** Potentially toxic elements, *Quercus ilex*, Post-mining, Geothermal, Urban, Tree leaves

## Abstract

In this study, leaves of the evergreen holm oak *Quercus ilex* were used to assess airborne contamination of potentially toxic elements (PTEs) at five towns located on the slopes of the Mt. Amiata (central Italy), an area with a long history of mining and, more recently, an important district for the industrial exploitation of geothermal energy. PTE composition and covariance of washed and unwashed *Q. ilex* leaves of three different ages (6, 12 and 24 month-old) were used to identify atmospheric inputs of PTEs at residential areas, evaluate long-term adsorption and retention of PTEs by the leaves, thus providing an indication of potential human exposure. Moreover, the determination of foliar concentrations of major elements (C, N, S and P) allowed an assessment of the nutritional status of the investigated urban tree stands which excluded the existence of stress condition caused by air pollution or other disturbances. Results indicated that overall Pb, Cu, and Cd concentration were low in the investigated urban sites, if compared with similar studies conducted in larger Italian cities, denoting a low contribution of vehicular traffic to the atmospheric pathway. The five urban settlements were characterized by a specific profile of elements (Al, Ba, Hg and Sb) enriched in unwashed leaves, resulting from the distinct geochemical characteristics of the area and from diffuse (i.e., urban activity) and point sources of PTEs emission (i.e., brownfields, geothermal power plants). The latter sources primarily govern the distribution of Hg, whose contamination was found to be very localized close to a major abandoned mining area. Our data provided quantitative evidence of the spectrum of PTEs potentially impacting resident population and may prove useful in support of follow-up instrumental monitoring campaigns of air quality, as well as for human health and ecological risk assessments.

**Graphical abstract:**

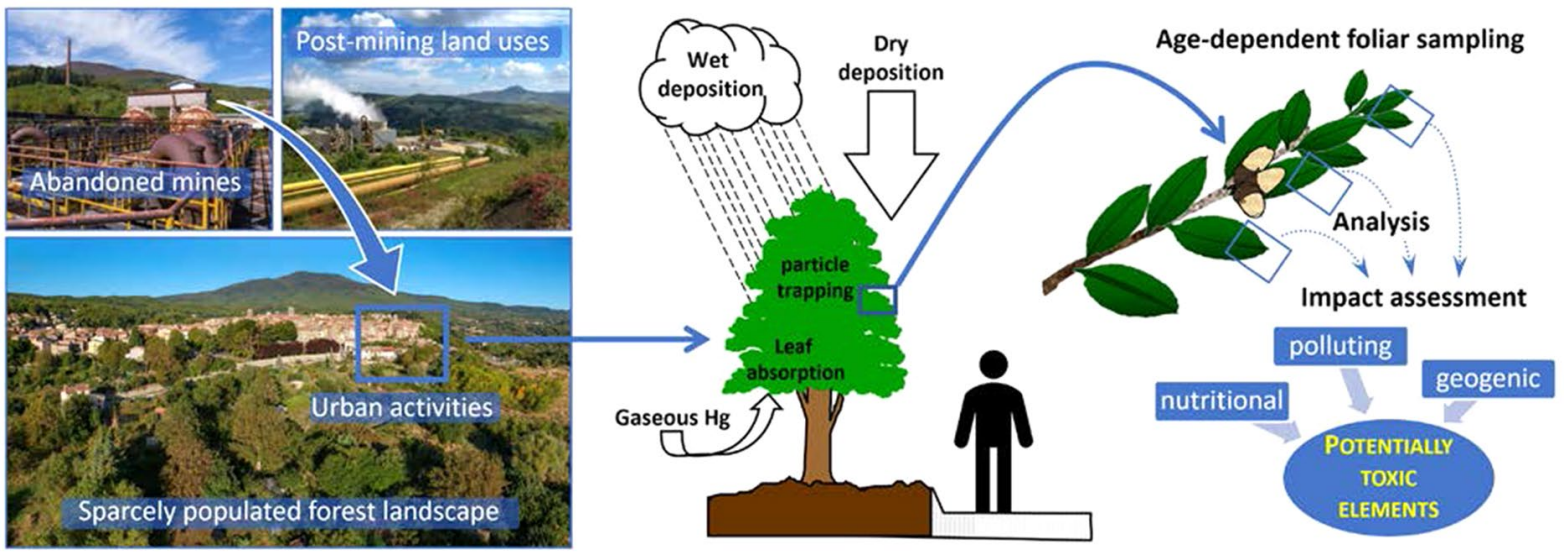

## Introduction

Mining is a global concern due to its impacts on vulnerable ecosystems and ultimately on human health (Luckeneder et al., 2021). Mining operations inevitably cause socio-economic consequences and produce several detrimental disturbances that commonly affect the surrounding environment. However, mining impacts can also have a global reach, as in the case of Hg, causing widespread health concerns for the general population and not only for high-end exposure individuals living in the vicinity of the mine sites (Gyamfi et al., 2021; Snow et al., 2021). Commonly, such impacts are not limited to the mine production phase but may also persist after mine closure and even rehearse over a longer time, if remediation measures are not adequate or absent at all (Monaci et al., 2021; Venkateswarlu et al., 2016). Ideally, mine reclamation should aim at returning land to a secure state, then ensuring public safety and allowing alternative land use possibilities to revitalize post-mining economy (Kivinen, 2017). Apparently, these aims have not been met in a vast number of mining areas all over the world, where mine closures were inappropriate or incomplete (Kretschmann, 2020). Consequently, a pressing need arises among policy-makers and risk assessors to achieve sustainable development targets in post-mining areas, which often presupposes taking the best advantage from any possible economic opportunity while properly managing contaminated lands with effective monitoring systems to protect human health (Gwenzi, 2021; Swartjes, 2015).

Potentially toxic elements (PTEs) include trace metals and metalloids (e.g., As, Cd, Pb and Hg) that can be released in the environmental media by a range of natural and anthropogenic sources (e.g. coal-fired power generation, waste incineration and mining; Klapstein et al., 2020). In urban and industrialized areas, human exposure to PTEs through contaminated air, water and soil has been connected with detrimental effects on health, including cardiovascular diseases, lung cancer, kidney failure and other organs disfunctions (Bini & Bech, 2014; Shaheen et al., 2020; Guarino et., 2021; Li et al., 2021). A main problem dealing with the monitoring airborne PTEs, a common issue in metal mining sites, is that human exposure in residential areas adjacent to the mine often remains undetermined, or not adequately assessed, due to the typically variable air concentrations in space and time, especially in topographically complex environments (Pappin et al., 2019; Wiseman et al., 2021). Post-mining areas are a special case in this respect as they are typically underdeveloped and often located in sparsely populated urban/rural landscapes (Kivinen, 2017). In such context, instrumental monitoring of PTEs in the air may be a complex task due to technical requirements (e.g., the need for electric power, analytical gases supplies) and high analytical costs that may limit the possibility to achieve adequate spatio-temporal coverage to capture the intrinsic variability of PTE contamination (Zalzal et al., 2020). Moreover, the overlapping of geogenic patterns and anthropogenic anomalies may complicate environmental risk assessment and entangle understanding of human health implications of enhanced PTEs circulation in post-mining environments (Guagliardi et al., 2013; Swartjes, 2011).

Leaves of suitable plant species have been commonly used to detect spatial and temporal enrichment of PTEs and other particulate matter (PM)-bound contaminants in urbanized contexts (Aničić Urošević et al., 2019; Baldantoni et al., 2020; Lin, 2015; Monaci et al., 2000; Terzaghi et al., 2020; Xiong et al., 2014). The capacity of certain perennial tree leaves to entrap PM and effectively collect atmospheric deposition of PTEs has found recent compelling scientific evidence (Chiam et al., 2019; Corada et al., 2021; Wang et al., 2015; Yin et al., 2019), which prompted further research to identify trees and other plants species to design residential green spaces having a beneficial contribution in mitigating airborne pollution in urban environments (Esposito et al., 2019; Han et al., 2020; Terzaghi et al., 2021).

In the present study, a set of PTEs and essential macroelements have been determined in holm oak (*Quercus ilex* L.) leaves, an evergreen broadleaf tree of the Mediterranean area. This tree species is of common use in urban gardens, parks and along roads as ornamental plant, and it has been found effective at monitoring and mitigating atmospheric PTEs and other contaminants associated with fine PM (< 10 µm in diameter), due to its favourable foliar traits (i.e., presence of trichomes, corrugated foliar surface, thick epicuticular waxes; Esposito et al., 2020; Monaci et al., 2000). The running hypothesis of this study was that the analysis of *Q. ilex* leaves for PTEs would allow to recognize the pattern of airborne PTEs in urban settlements of the Mt Amiata, a post-mining area in central Italy partially converted as geothermoelectric district, and thereby to support the identification of potential pathways of exposure to PTEs for the resident population. To investigate the leaf capacity to hold entrapped PM-bound PTEs, the analytical data from unwashed and washed samples were compared, according to an established technique in biomonitoring by *Q. ilex* leaves (De Nicola et al., 2008; Esposito et al., 2019; Ugolini et al., 2013). Additionally, nutritional status (e.g., C, N, P, S) was also assessed by foliar analysis to highlight eventual condition of stress in tree stands at urban sites that can be related to the atmospheric pollution (Baldantoni et al., 2020; Bulbovas et al., 2020; Maisto et al., 2013). The elemental composition of the foliage of holm oak was investigated in relation to different age classes of washed and unwashed leaves with the specific aims of: (1) assessing PTEs concentrations changes among age groups to identify potential accumulation of specific elements; (2) defining pattern of PTE enrichment in the urban sites with respect to the proximity of potential sources of contamination; (3) highlighting nutritional element interactions or imbalances in plants reflecting impacts from atmospheric pollution or other anthropic disturbances.

## Materials and methods

### Study area

The Mt. Amiata is in the Tuscany region (central Italy), about 130 km North-Northeast from Rome and 100 km South-Southwest from Florence. It is an isolated summit (1738 m) whose natural arboreal vegetation is characterized mainly by beech (*Fagus sylvatica*), silver fir (*Abies alba*), chestnut (*Castanaea sativa*) located at different altitudes and exposures (Selvi, 1996). The forest landscape in the Mt. Amiata is punctuated by few villages and towns where the holm oak (*Q. ilex*) is often present as ornamental tree at roadsides and urban parks.

In addition to orographic isolation, the most distinct characteristic of the Mt. Amiata is its recent volcanic origin. This underly a well-known Hg mineralization (cinnabar, HgS), which has been mined since Etruscan time (900-700 BC). Modern mining operation in the Mt. Amiata started in 1846, and since then, several mines were established, mainly on the southern and eastern flanks of the mountain. The mine of Abbadia San Salvatore (42.87° N, 11.66° E; 822 m a.s.l.), provided with an on-site metallurgical facility, was the largest in the region and remained among the global leading Hg producers for most of the twentieth century (McLagan et al., 2019; Vaselli et al., 2017). Even before the closure of this last mine in 1984, a complex phase of redevelopment had started, which partly converted the territory towards geothermoelectric power production (Bacci et al., 2000).

At the end of 1960s, when mining industry of the Mt. Amiata was already experiencing a profound crisis, new economical enterprises were undertaken for the exploitation of the geothermal energy sources of this area for power production. Since the beginning, however, this industry aroused concern among the resident population, due to the anomalous levels of Hg, S and various PTEs (i.e., As, Sb) in the geothermal fluids (Bravi & Basosi, 2014). Presently, there are six geothermal plants in the Mt. Amiata, accounting for an overall nominal capacity of 120 MW. All the geothermal plants in the area use the flash technology which is characterized by the emission of non-condensable gases and trace elements, whose concentrations can range widely (Mutia et al., 2016).

Health status of the populations residing in geothermal districts of Mt. Amiata is a matter of concern for the regional public health authorities, due to epidemiological anomalies in mortality and morbidity rates that have emerged within the local population in the past (Minichilli et al., 2012). More recently, targeted studies conducted in the Mt. Amiata residents reported excess risk for respiratory diseases and other non-accidental and cause-specific mortality and morbidity (Bustaffa et al., 2020; Nuvolone et al., 2019; Profili et al., 2018).

### Sampling and sample preparation

Leaves of *Q. ilex* were collected between 5 and 8 April 2017 at five urban areas of the Mt. Amiata where Hg mining and/or industrial exploitation of geothermal energy were present: Abbadia San Salvatore (Abb; population: 6150), Arcidosso (Arc; 4295), Bagnore (Bag; 395), Piancastagnaio (Pia; 4923) and Santa Fiora (San; 2611). At each station, geographical coordinates, elevation and other features (e.g., proximity to high buildings) of the site were noted, and from three randomly selected trees, three terminal portions of the branches, each from different parts of the crown, were excised at a height of 2.5–3.5 m from the ground. The selected material was immediately processed for eliminating unnecessary or senescent twigs and leaves, and eventual foreign material. The material was stored in a single large paper wrapper identified by the date of collection, location and the station number. The same procedure was also applied in a remote area in Tuscany, 15 km East of Siena, far from known sources of pollution, where sampling of *Q. ilex* leaves was replicated at three sites, representing control areas reflecting the regional baseline of elemental concentrations.

At the laboratory of the University of Siena, intact, healthy, and dimensionally homogeneous foliage was selected from the sample formed in the field and leaves were excised, classified by their phyllotaxis (i.e., considering the leaf position on the branch, its degree of lignification, hairiness) and grouped into three age classes (approx. 6, 12 and 24 months; Bargagli, 1998; Monaci et al., 2000) and then dried at room temperature. Half of each holm oak sample (about 150 g) was subjected to washing to remove atmospheric particulates adsorbed to leaves by thoroughly rinsing the leaves with deionized water, since *Q. ilex* leaves are known for being an efficient collector of atmospheric particulate matter (De Nicola et al., 2013, 2015; Monaci et al., 2000; Muhammad et al., 2020). Each leaf was subjected to three washing cycles of five-six seconds followed by drying with tissue paper and, finally, at room temperature. The resulting dried plant material was pulverized and homogenized in a centrifugal ball mill (MM2000, Retsch GmbH Haan, Germany); then, it was placed in an airtight PVC container and kept in the laboratory until analysis was performed at the University of Hohenheim (Stuttgart, Germany).

### Target elements and analysis

Target analytes included nine potentially harmful elements (Al, Ba, Cr, Fe, Sb, Cu, Ni, V and Zn) and four elements of widely recognized toxicity (As, Cd, Pb and Hg; Bini & Bech, 2014; Li et al., 2021). Supplementing PTEs were the other six major elements (C, K, Mg, N, P and S) of nutritional relevance in plants (Kabata-Pendias & Mukherjee, 2007).

For the analysis, 0.4 g aliquot of each homogenized sample was weighted in a polytetrafluoroethylene (PTFE, Teflon®) container; then, after adding 5 mL of concentrated HNO_3_ (65% JT Baker, analytical grade) and 2 mL of H_2_O_2_ (20%, JT Baker, analytical grade), the PTFE containers were hermetically sealed and put inside in a microwave UltraCLAVE III digestion unit (MLS GmbH, Leutkirch, Germany) for mineralization of the sample according to a specific program at controlled temperature (max 210 °C) and pressure (max 160 bar). One reagent blank (only HNO_3_ and H_2_O_2_ in a container) and 0.4 g of the standard reference materials 1573a “Tomato Leaves” and 1547 “Peach Leaves” from NIST (Gaithersburg, USA) were included in each batch of analysis (*n* = 24 samples) and subjected to the same analytical procedures as the samples. In case the elemental concentrations of the reference materials did not fall within the certified concentration and uncertainty, the mineralization and analytical determinations of the samples were repeated.

At the end of the mineralization program, the PTFE containers were allowed to cool before their opening and the mineralization solutions were transferred to a polyethylene (PE) tube inside a suction hood, avoiding any leakage or contamination, and made up to the final volume of 10 mL with Milli-Q deionized water (Millipore®). Instrumental calibrations were performed using aqueous reference solutions prepared immediately before use from serial dilution from commercial stock solutions (Merk, Germany) at a concentration of 1 g/L. The determination of the total concentrations of major elements and PTEs was carried out, according to the type of elements and the expected analytical levels in the samples. An inductively coupled plasma optical emission spectrometer (ICP-OES, 5110 SVDV, Agilent, Santa Clara, USA) was used for the determinations of Al, Ba, Cu, Fe, K, P, Zn; an inductively coupled plasma mass spectrometer (ICP-MS, NexION 300X, PerkinElmer, Rodgau, Germany) was used for As, Cd, Cr, Hg, Ni, Pb, V, and Sb; an element analyzer (EA, vario EL CUBE, Elementar Analysensysteme GmbH, Hanau, Germany) was used for the determination of C, N and S, by weighting an aliquot of 15–30 mg of the milled samples or the standard reference materials into specific tin cups of an autosampler. As a part of the analytical quality control, standard solutions were analysed after every 12 samples; for each batch of analysis (*n* = 24), replicate and procedural blanks determinations were carried out to evaluate sample homogeneity and uncertainties due to the mineralization. Analytical precision in the leaf samples ranged from 2 for Cd to 21% for As and detection limits (ranging from 0.5 to 21 ng/L) were estimated on the basis of the standard deviations of blanks triplicates.

### Data analysis

Normality of distributions and homogeneity of variance of the element concentrations dataset were assessed using the Shapiro–Wilk’s test and the Levene’s test. As some elemental distributions were asymmetric and the assumptions of normality and homoscedasticity were not always met, robust descriptors of central tendency and variability (median and median absolute deviation, MAD) were also used. On standardized data, the analysis of variance and the Scheffé test were performed to test differences in elemental concentrations between washed and unwashed leaves of different ages and sampling areas. Relationships among element concentrations in leaves were determined using Pearson’s correlation coefficients (*r*) calculated on normalized dataset which were used also to measure the degree of similarity of element concentrations at the municipality levels by performing discriminant analysis (DA). Two discriminant variables were extracted which represented the differential trace element enrichment in the five urban environments of this study. Mahalanobis distances of the centroid of each habitat group were tested for differences. The statistical analyses were performed by R software (R Core Team, 2020).

## Results

### Foliar element concentrations

Table 1 reports overall average concentrations of major and trace elements in *Q. ilex* leaves from five small urban areas of the Mt. Amiata. In the table, descriptive statistics are reported for each element along with reference data of the same species from urban or mining environments. A control area in Tuscany distant from known sources of PTE contamination, reflecting regional baseline of elemental concentrations in *Q. ilex* leaves, is also included in the table for comparisons. Overall, dispersion of foliar elemental concentrations expressed as coefficient of variation (CV; Table 1) remained lower than 100%, with Hg being the most variable among the investigated elements (CV = 98%) while most of other PTEs (Al, As, Ba, Cr, Cd, Fe and Pb) had CVs ranging between 45 and 73%. The corresponding figures of PTEs from *Q. ilex* leaves of other, larger urban areas in Italy, for which comparisons are possible (i.e. Al, Cd, Cr, Cu, Ni, Pb, Zn), were generally similar (e.g., for Ni, Cr) to or mainly higher (i.e., Cu, Zn) than those of the present study. The literature data for As, Hg, and Sb in *Q. ilex* leaves are not available or scanty for urban environments and mostly limited to mining sites reflecting condition of high pollution levels. Ranges of variation in concentrations of major elements were limited in the study area, with the lowest CVs (2%) found for C (Table 1).

**Table 1:** Basic statistics of PTEs and major element concentrations (mg/kg or g/kg DM, as indicated; *n* = 39) in unwashed leaves of *Q. ilex* from five urban sites of M. Amiata and at rural/forested areas of Tuscany (*n* = 3) reflecting unpolluted conditions and regional baselines of foliar element concentrations of *Q. ilex*. Reference data from Italian urban sites and mining sites from Spain are reported

Data	Areas	Statistics/reference	Al	As	Ba	Cd	Cr	Cu	Hg	K	Mg
			mg/kg								
This study		Mean	309	0.14	23.0	0.04	1.06	4.64	0.14	6108	860
		Median	255	0.13	18.1	0.03	0.88	4.57	0.08	6196	841
	Urban	Standard deviation	184	0.07	16.2	0.01	0.55	0.74	0.14	1008	308
	Mt. Amiata	Median absolute deviation	98.6	0.05	8.51	0.00	0.30	0.65	0.03	646	217
		Coefficient of variation (%)	59.5	50.5	70.4	30.6	52.1	16.0	98.3	16.5	35.8
		Minimum	122	0.04	6.81	0.03	0.28	3.39	0.03	3987	347
		Maximum	929	0.34	80.5	0.07	2.75	6.61	0.56	7966	1776
	Rural	Mean	272	0.13	23.9	0.07	1.33	4.06	0.05	6085	959
	Tuscany	Standard deviation	104	0.05	12.9	0.02	1.00	0.52	0.01	5495	163
Reference		Alfani et al. (2000)	–	–	–	0.16	0.69	16.1	–	–	–
		De Nicola et al. (2003)	–	–	–	0.06	0.26	3.90	–	7130	1300
	Urban	Ugolini et al. (2013)	–	–	35	0.12	3.10	19.8	–	–	–
	Italy	Maisto et al. (2013)	–	–	–	–	–	10.0	–	7000	1500
		Fantozzi et al. (2013)	416	–	–	0.03	2.52	10.9	–	4894	1214
		Baldantoni et al. (2020)	375	0.01	–	0.07	1.11	7.89	–	6568	2220
	Mining sites	Higueras et al. (2017)	–	0.56	–	–	–	5.19	19.7	–	–
	Spain	Rodríguez Martín et al. (2015)	265	–	–	2.13	14.6	10.2	–	5600	2400

Data	Areas	Statistics/reference	Ni	P	Pb	Sb	Zn	C	N	S
			mg/kg					g/kg		
This study		Mean	0.82	987	0.44	0.08	22.6	46.8	1.40	0.10
		Median	0.63	857	0.39	0.08	19.9	47.0	1.38	0.10
	Urban	Standard deviation	0.61	435	0.21	0.03	8.42	0.72	0.23	0.02
	Mt. Amiata	Median absolute deviation	0.23	106	0.15	0.03	3.33	0.40	0.15	0.01
		Coefficient of variation (%)	74.3	44.0	47.8	38.4	37.2	1.50	16.4	17.6
		Minimum	0.22	650	0.16	0.05	11.4	45.2	0.97	0.07
		Maximum	2.99	2937	0.98	0.16	46.1	48.0	1.96	0.13
	Rural	Mean	2.77	950	0.62	0.05	25.9	47.4	1.41	0.12
	Tuscany	Standard deviation	2.29	0.02	0.24	0.01	7.30	0.41	0.18	0.04
Reference		Alfani et al. (2000)	1.40	–	5.31		48.0	50.6	1.93	0.13
		De Nicola et al. (2003)	0.53	–	0.40		30.0	–	–	–
	Urban	Ugolini et al. (2013)	–	–	3.70		42.8	–	–	–
	Italy	Maisto et al. (2013)	–	1400	–		35.0	49.0	1.6	0.11
		Fantozzi et al. (2013)	1.15	1075	0.88		34.1	–	–	0.14
		Baldantoni et al. (2020)	0.16	1161	0.29		22.4	–	–	0.22
	Mining sites	Higueras et al. (2017)	–	–	131		165	–	–	–
	Spain	Rodríguez Martín et al. (2015)	10.6	800	3.14		29.9	–	–	0.13

**Fig. 1:**
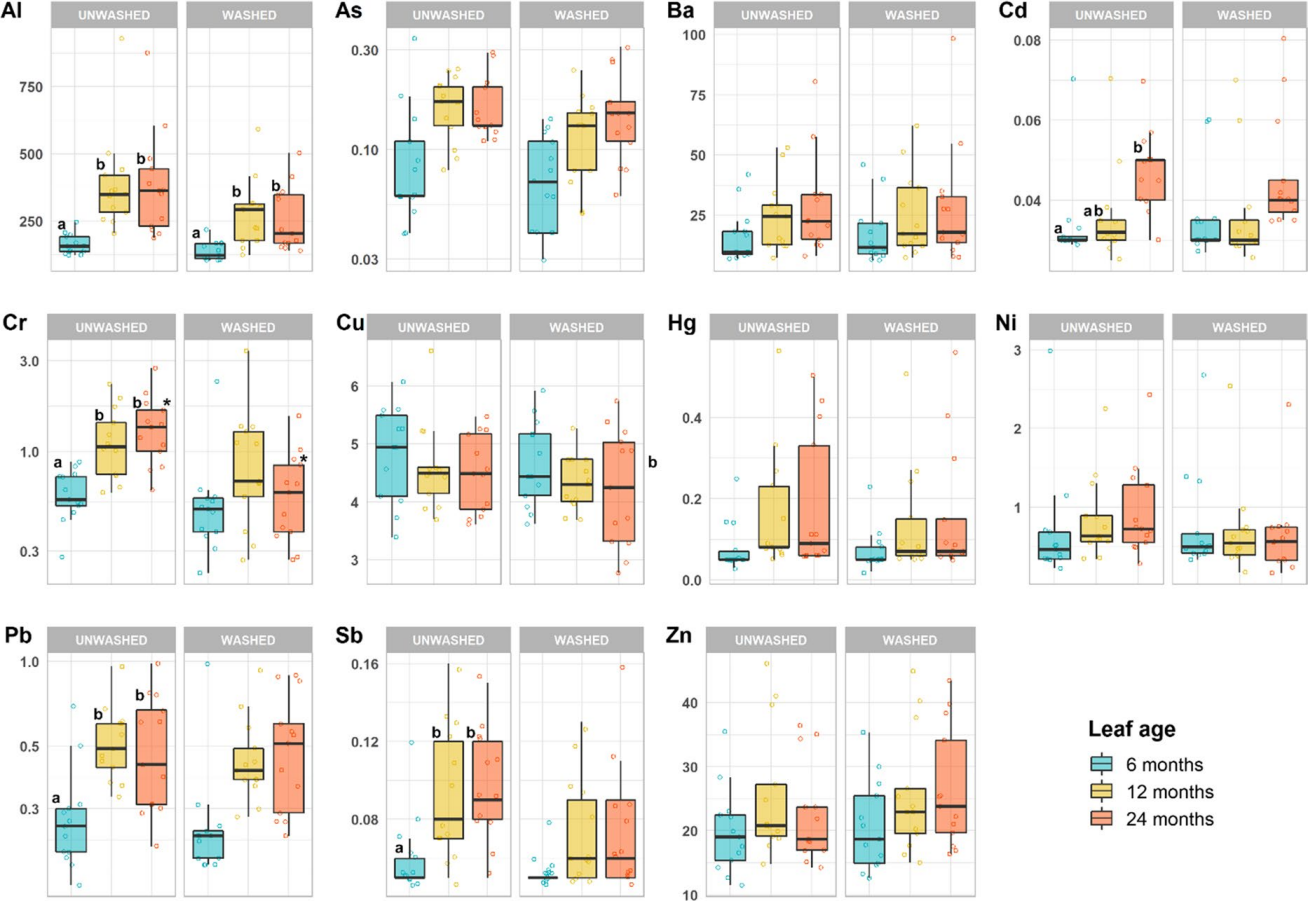
Concentrations (median, 1st and 3rd quartile, min–max; mg/kg, DM) of PTEs in washed and unwashed 6, 12, 24 month-old *Q. ilex* leaves from urban areas of the Mt. Amiata. Different letters indicate statistical differences (*p* < 0.01) among age groups (Scheffé test on normalized data); asterisks indicate differences (*p* < 0.01) between washed/unwashed groups of the same age

A general increasing trend of PTE concentrations with the age of unwashed leaves (6, 12, and 24 month-old) is noticed for Al, As, Ba, Cd, Cr, Fe, Hg, Pb, Sb, V and Zn (Table 1). Significant differences (*p* < 0.01) among the leaf age groups were found for Al, Cd, Cr, Sb. Commonly, the latter elements showed significantly lower concentrations in the young leaves (6 month-old) than in the more mature leaves (12 and 24 month-old). For most PTEs, the effect of washing was not significant and only a slight decrease in concentrations was generally observed in washed leaves with respect to unwashed ones (Fig. 1). Statistically significant (*p* < 0.01) differences between washed and unwashed leaves were found only for Cr in 24 month-old leaves.

**Fig. 2:**
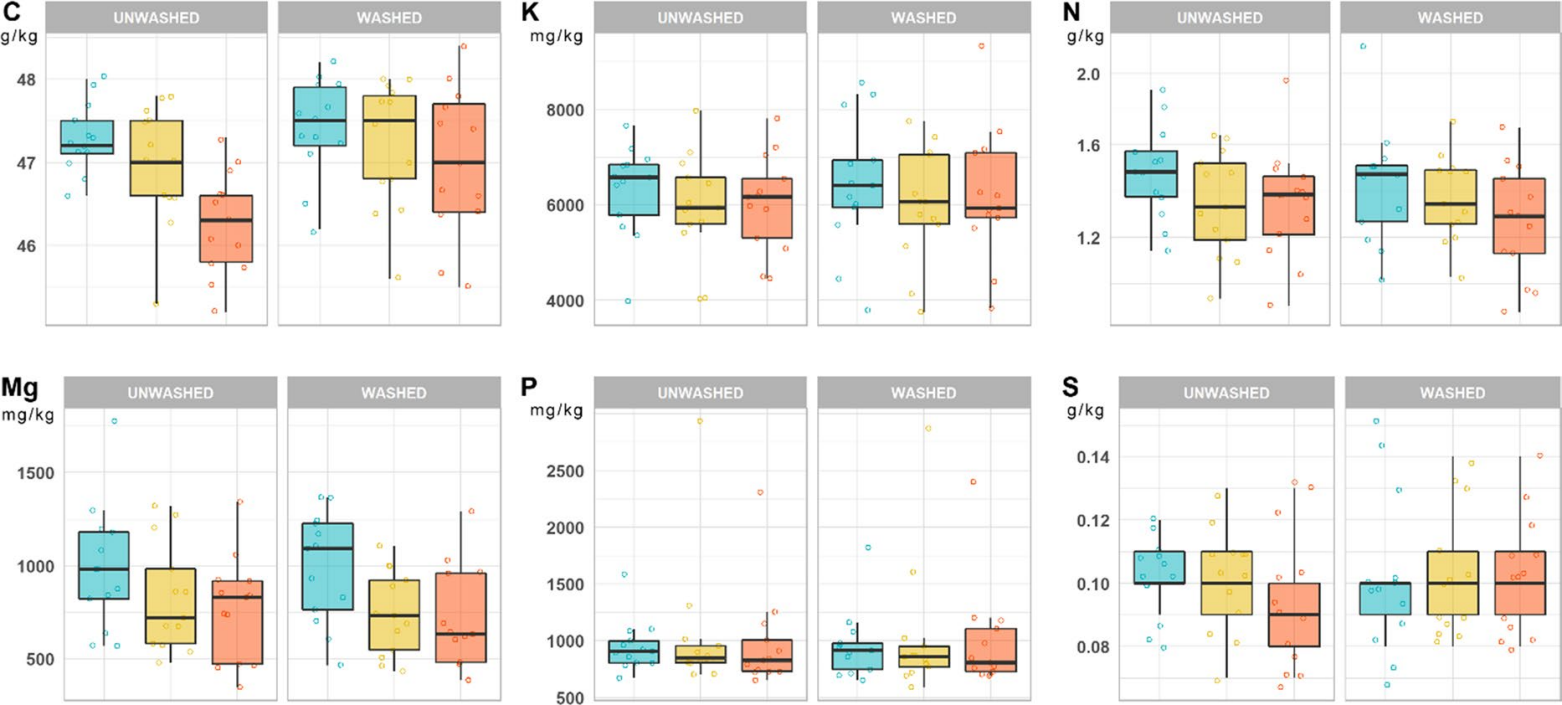
Concentrations (median, 1st and 3rd quartile, min–max; mg/kg or g/kg, DM) of macronutrients in washed and unwashed 6, 12, 24 month-old *Q. ilex* leaves from urban areas of Mt. Amiata. No statistically significant differences (*p* < 0.01) were found among leaf-age groups of washed and unwashed leaves and between washed/unwashed leaves of the same groups

Overall, a decreasing trend with the ageing of the leaves was recognized for C, Cu and Mg (Fig. 2), but no significant differences were found among any of the investigated macronutrients with respect to the leaf age groups. Also, washing of the leaves was not associated to any significant change in macronutrient concentrations (Fig. 2). The latter corresponded well to the normal ranges for the healthy growth of plants (Knecht et al., 2004; Marschner, 2012; Watanabe et al., 2007) and were not significantly correlated with PTEs.

**Fig. 3:**
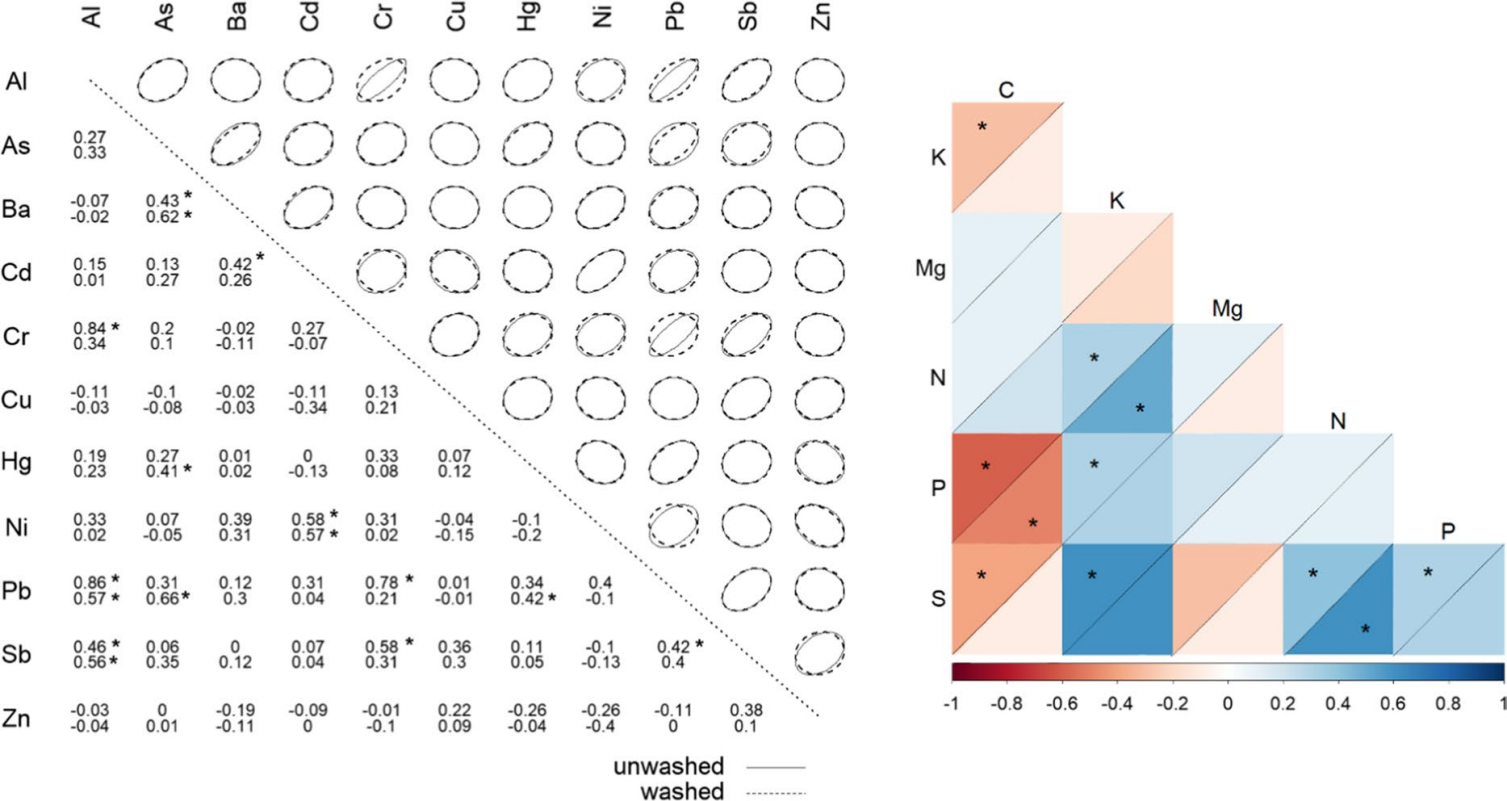
Left: correlation matrix of PTEs in unwashed and washed (below, dotted line) *Q. ilex* leaves. Right: correlation matrix of macronutrients in unwashed (above the diagonal) and washed (below) *Q. ilex* leaves. The asterisk indicates statistically significant correlations (*p* < 0.01)

In unwashed *Q. ilex* leaves, Al, Cr, Pb and Sb were all positively intercorrelated (*p* < 0.01). Significant correlations were also found for As vs. Ba and Cd vs. Ni (*p* < 0.01; Fig. 3, left inset). PTEs accumulation of washed leaves generally showed fewer, less significant patterns of reciprocal correlations, especially those with Cr (i.e., Al vs Cr, Pb vs Cr). Notably, significant correlations (*p* < 0.01) of As with Hg and Pb were found in the washed leaves. Only to a very limited extent, correlations among macronutrients (i.e., C vs K, C vs P, C vs S and K vs N) were affected by washing (Fig. 3, right inset). Carbon, P, and S were positively intercorrelated, while N, P, K and Mg were generally distinguished by mutual inverse correlations.

**Fig. 4:**
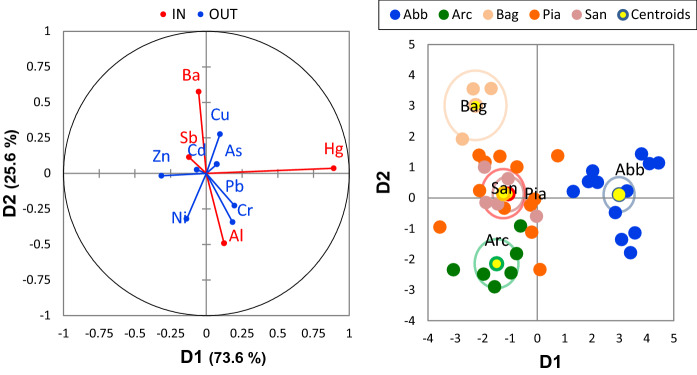
Discriminant analysis of PTEs concentrations in unwashed *Q. ilex* leaves from five urban settlements of the Mt. Amiata (Abb, Arc, Bag, Pia, San)

**Fig. 5:**
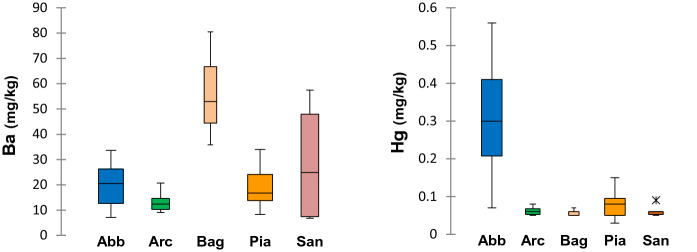
Box and whisker plots of Ba (left) and Hg (right) concentrations (mg/kg, DM; *n* = 39) in unwashed *Q. ilex* leaves of five urban settlements (Abb, Arc, Bag, Pia, San) of the Mt. Amiata

Main results of the discriminant function analysis performed on the dataset of the PTEs concentrations in unwashed leaves are reported in Fig. 4, assuming the town of sampling as the grouping variable. Four eigenvalues of roots were extracted from the computation, and the first two discriminant roots were highly significant (Bartlett’s test of eigenvalues *p* << 0.01) and accounted for practically all (99.1%) of the original variance of data. By adopting a stepwise approach, Hg, Ba, Al, and Sb were, in the order, included in a model (Fig. 4, left inset) which discriminated PTE composition and covariance of the leaves of *Q. ilex* according to their provenance (Fig. 4, right inset). Discriminant functions based on the Hg, Ba, Al, and Sb foliar concentrations effectively predicted the origin of the leaves from the five towns in the study area. The first discriminant root was governed by Hg variance whose concentrations in *Q. ilex* leaves were especially high at Abbadia San Salvatore (Abb; Fig. 5), while the most significant contribution to the second root was by Ba, whose concentrations were higher in Bagnore (Bag; Fig. 5).

## Discussion

In this study, holm oak leaves from five towns located in the eastern, southern, and western slopes of the Mt. Amiata were generally characterized by low levels of PTEs, if compared to other similar studies carried out in much larger and populated urban agglomerations (Table 1). Comparison between PTEs datasets of washed and unwashed leaves did not reveal considerable load of typically geogenic elements from resuspended dust onto the leaves, a circumstance which is commonly observed in trafficked urban environments (Fang et al., 2021; Memoli et al., 2020). Under laboratory and field conditions, holm oak leaves have been found particularly effective at capturing airborne PM-bearing metals and rather well, among other common species in Mediterranean urban environments, at retaining the particles on the leaf surface (Blanusa et al., 2015; Fantozzi et al., 2013). In this regard, the evergreen broadleaves fitted with trichomes and epicuticular waxes are the attributes that make *Q. ilex* useful for biomonitoring atmospheric contamination, even in comparison to tree species of the same genus (e.g., *Q. cerris*, *Q. robur*; Aboal et al., 2004; Blanusa et al., 2015). It is noteworthy, however, that elements commonly emitted by vehicular traffic, such as Cd, Pb, Zn and Cu originating from exhaust emissions and/or wearing of vehicle components (i.e., brakes, tyres; Wawer et al., 2020; Wiseman et al., 2021), were not found considerably enriched in the leaves of different age, hence not revealing any significant foliar accumulation of these elements from atmospheric deposition over a long period of exposure (12, 24 months). This result is coherent with the features and the remoteness of the investigated urban sites and it is further corroborated by the good nutritional status revealed by the *Q. ilex* leaves which remained unaltered across the study area. Foliar concentrations of essential major elements (i.e., C, N, P and Mg) of *Q. ilex* of the Mt. Amiata practically coincided with those measured in stands of the same species from the control area of this this study (Table 1) and from a pristine forest ecosystem in Catalonia (Sardans et al., 2011). Moreover, two specific indicators of the nutritional status of *Q. ilex,* K and Mg (De Nicola et al., 2003) showed concentrations in the leaves of this study well within the range of normality. Nutritional unbalance in leaves is considered a common diagnostic stress symptom in polluted urban and peri-urban areas (Bulbovas et al., 2020). Excess of metal accumulation was found responsible for nutritional syndromes and other physiological impairments in *Q. ilex* leaves from the trafficked Naples, in Italy (Arena et al., 2017; Maisto et al., 2013). More recently, Fusaro and co-authors ranked *Q. ilex* as comparatively resistant to urban pollution and specifically capable of adaptive stress response to low/intermediate PM deposition (Fusaro et al., 2021).

Multivariate analysis (DA) applied to the *Q. ilex* foliar concentrations dataset identified the PTEs contamination in the study area as primarily characterized by the enrichment in Hg, Ba, Al, and Sb (Fig. 4). The multielemental pattern recognized in this study is rather unique and denotes the distinct environmental settings of Mt. Amiata, entangling geogenic (geochemical anomaly, natural geothermal activities) and possible man-made sources of PTEs (related to mining, urban activity, and/or the industrial exploitation of geothermal energy). Among the above-mentioned elements outlined by DA, Hg did not come as a surprise, as the enrichment of this metal in environmental and biological matrixes (e.g., air, soil, and plants) has been already well documented in the Mt. Amiata area as an indication of enhanced geosphere-biosphere circulation of the metal (Chiarantini et al., 2016; Protano & Nannoni, 2018; Vaselli et al., 2013). Throughout the industrial history of this region, different mines were opened and put into operation, encountering alternating fortunes in the global markets of Hg production (Strappa, 1977). When in operation, these sites caused massive and prolonged discharges of gaseous elemental Hg (GEM) in the local and global atmosphere (Vaselli et al., 2013). Even now, the contaminated site of the historical mine located in the direct proximity of the residential area of Abbadia San Salvatore (Abb), the largest ever put in operation in the Mt. Amiata and presently in the process of reclamation, still represents a significant source of emission estimated in 80 and 150 kg GEM/year in the cold and hot seasons, respectively (McLagan et al., 2019). GEM uptake occurs in vascular plants through foliage, by cuticular and stomatal absorption (Esbrí et al., 2018; Higueras et al., 2017). Data of the present study, based on foliar Hg accumulation, indicate three times higher average Hg concentrations in Abb than in the other towns of the study (Fig. 5). The comparatively high variability of Hg concentrations determined in Abb (CV = 50%, Fig. 5) is indicative of wide array of concentration within the urban area, in relation to the proximity to the mine site, as emerged from different campaigns of GEM concentration monitoring carried out in the past at the mine (McLagan et al., 2019; Vaselli et al., 2013).

Geothermal power production using flash technology, the current standard for high pressure, water dominated geothermal reservoirs such as that of the Mt. Amiata (Bravi & Basosi, 2014), is a known source of Hg and other PTEs emission in the atmosphere (Mutia et al., 2021; Parisi et al., 2019). However, the present study did not find any evidence of enrichment of Hg or other toxic elements commonly associated with geothermal sources, such as As or Sb, in *Q. ilex* leaves from the urban areas of Bagnore and Piancastagnaio, each hosting three geothermal power plants with the same nominal power (20 MW; ARPAT, 2021). The concentrations of As in *Q. ilex* leaves of Mt. Amiata urban areas always fell within the normal range of plants growing in unpolluted areas (0.005–0.080 mg/kg, Kabata-Pendias, 2011), while average foliar concentrations of Sb showed only a slight anomaly with respect to those found in rural areas of Tuscany, taken as control reference (Table 1). The only possible load of PTE attributable to the geothermal emissions can possibly be found, among the elements of this study, in the relatively high concentrations of Ba in the unwashed leaves of Bagnore (Bag; Fig. 5), little village located downwind with respect to the homonymous geothermal district. Such as foliar Ba enrichment could result from the atmospheric deposition of barite (BaSO_4_), which is a tracer for the geothermal brine and can be released in air with steam condensate during geothermal power production (Finster et al., 2015; Orywall et al., 2017; Tranter et al., 2020).

The results of the present study seem to conflict with those of other biomonitoring studies carried out in the Mt. Amiata area in the past (Bacci et al., 2000; Bargagli et al., 1997; Paoli & Loppi, 2008) that linked contamination of local vegetation by Hg and other element (e.g., As, Sb, and S) to the emissions from the geothermal power plants present in the area. Two main reasons may be addressed to explain such as discrepancy of results. Firstly, the present study was aimed at showing potential impact of PTEs in urban areas while previous studies were mostly performed in forested/rural environments at close distance to the geothermal plants. The second reason refers to the important technological improvements for the reduction of emissions that have been implemented at all geothermal plants of the Mt. Amiata area over the last decade. For example, a main piece of technology that presently equips all these power plants is AMIS®, developed and patented by the Italian National Electricity Authority (ENEL), allowing for a drastic reduction of Hg and H_2_S emissions (Bustaffa et al., 2020; Nuvolone et al., 2020). Although this and other technological advancements have substantially contributed to contain environmental impacts of this type of energy production on the local air quality (Parisi et al., 2019), still there are health concern among residents and the regional and local health authorities that recently prompted epidemiological and human biomonitoring studies targeting PTEs (Bustaffa et al., 2020; Nuvolone et al., 2019; Profili et al., 2018).

## Conclusions

The present study was based on *Q. ilex,* a tree species commonly used in the Mediterranean area for building urban green infrastructures and performing biomonitoring studies. The foliar elemental composition of this tree species, investigated in relation to different age classes of washed and unwashed leaves, allowed to define the pattern of airborne PTE contamination across different towns of an historical mining region, transitioning to a new economic framework, not without controversies and environmental concerns. The determined contamination profile was mainly characterized by Al, Ba, Hg and Sb, which reflected both the peculiar geolithological settings of the studied area and the anthropogenic disturbances therein. Most notably, our data point out the highest Hg contamination in correspondence of a disused mine site, a result which stresses the need of follow-up studies to ascertain the effective health impacts of this source on the resident population, and ultimately put emphasis on the urgence of the completion of the clean-up operations at this derelict mine site. From the methodological point of view, the present study proved the effectiveness of biomonitoring by foliar analysis in providing quantitative information on airborne PTEs contamination in sparsely populated areas, such as that of this study. Much of this information would have been undetected by using an instrumental monitoring approach due to economic and technical constraints. Finally, based on the proof that the measured PTE foliar concentration data are indicative of the respirable fraction, *Q. ilex* leaves are effective sampling media to identify those PTEs that are most likely to represent a potential human exposure and can serve as ground to set up targeted instrumental air quality monitoring, as well as human health and ecological risk assessments.
