# Correction to: Air quality in post-mining towns: tracking potentially toxic elements using tree leaves

**DOI:** 10.1007/s10653-022-01273-1

**Published:** 2022-04-13

**Authors:** Fabrizio Monaci, Stefania Ancora, Luca Paoli, Stefano Loppi, Jürgen Franzaring

**Affiliations:** 1 Department of Life Sciences, University of Siena, Via Mattioli 4, Siena, Italy; 2 Department of Physical Sciences, Earth and Environment, University of Siena, Via Mattioli 4, Siena, Italy; 3 Department of Biology, University of Pisa, Via Luca Ghini, 13, 56126 Pisa, Italy; 4 Institute of Landscape and Plant Ecology, University of Hohenheim, Ottilie-Zeller-Weg 2, 70599 Stuttgart, Germany

## Correction to: Environ Geochem Health https://doi.org/10.1007/s10653-022-01252-6

In the original publication of the article, the first and last name of the fourth author was incorrectly published as “Loppi Stefano”. The correct name is “Stefano Loppi”.

The original article has been corrected.

